# Connected multi-vehicle crash risk assessment considering probability and intensity

**DOI:** 10.1371/journal.pone.0313317

**Published:** 2025-02-21

**Authors:** Shuo Jia, Jin Xu, Song Wang, Xingliang Liu

**Affiliations:** College of Traffic & Transportation, Chongqing Jiaotong Unversity, Chongqing, China; Nanjing Forestry University, CHINA

## Abstract

Accurate driving risk assessments are essential in vehicle collision avoidance and traffic safety. The uncertainty in driving intentions and behavior, coupled with the difficulty in accurately predicting future trajectories of vehicles, poses challenges in assessing collision risk among vehicles. Existing research on collision risk assessment has been limited to focusing on pre-crashes (e.g., time-to-collision) and ignoring the impact of crash severity on risk. Research integrating pre- and post-crash is needed to assess the collision risk comprehensively. Therefore, the objective of this study was to propose an assessment model for collision risk in a vehicle-to-vehicle communication environment to achieve a more scientific assessment of driving risk by integrating probability (pre-crash) and intensity (post-crash). The proposed trajectory prediction model takes driving intentions into account and employs a social tensor pool to integrate interactions between vehicles, thereby achieving improved prediction accuracy. The likelihood of collision is obtained by analyzing the conflict relationship between the predicted and candidate trajectories of different vehicles. This study proposes a risk assessment model comprising two parts: one assesses the likelihood of collision by analyzing the conflicted relationship between predicted and candidate trajectories of different vehicles, and the other determines collision intensity through analysis of vehicle driving states. Finally, publicly available unmanned aerial vehicle (UAV)-based traffic data are used to validate the models. The prediction errors of the proposed trajectory prediction model for three-second trajectories are 0.68 m and 1.34 for the root mean square error and negative log-likelihood, respectively. The quantitative experimental results illustrate that the proposed model outperforms existing models and can scientifically assess the risk of vehicle travel.

## Introduction

The development of advanced driving assistance systems (ADAS) effectively reduces the burden on drivers, and ADAS-equipped intelligent connected vehicles (ICVs) have unique advantages in terms of environmental perception and vehicle operation. Over the past few years, the ICV penetration has increased. In China, the penetration rate of vehicles with ADAS has reached 22.58% in 2022. Estimates for the United States suggest a projected market penetration rate of 40% to 48% by 2035 [1]. Simultaneously, there has been a significant increase in traffic accidents or issues caused by ADAS, and the reliability and safety issues of ICVs have become the focus of attention for the automotive market and researchers [2, 3].

Inaccurate assessment of driving risks is a primary reason for the frequent occurrence of vehicular traffic accidents. An ADAS mainly relies on sensors to perceive the driving environment, uses risk assessment models to determine a collision risk in the current driving state, and controls the vehicle or reminds the driver to avoid such risks. An accurate risk assessment model is a key technology for the safe driving of ICVs and AVs [4]. Risk assessment results prevent vehicle collisions and provide critical information for autonomous driving [5–7].

Research on risk assessment has been a hot topic in the field of transportation safety. Early models were frequently grounded in vehicle kinematics and dynamics theory and incorporated into automatic emergency braking (AEB) systems. Assessment results inform vehicle or driver operations, facilitating the maintenance of safe distances through throttle and brake control in longitudinal motion [8, 9]. Additionally, they aid in avoiding collisions with adjacent vehicles in lateral motion by steering wheel angle adjustments. These models evaluate collision risks based primarily on distance and time measures [10], employing methodologies such as time-to-collision (TTC) [11, 12], time-to-brake, time-to-react (TTR), and time headway (THW) [13]. TTC is the most commonly used model because of its simplicity and rationality. Furthermore, TTC can be computed as the root of polynomials interpolated between extended entities, thereby enhancing the calculation efficiency and precision [14]. [15] introduced concepts and calculation methods for the longitudinal and lateral collision risks of vehicles based on the traditional time–distance model and integrated the mechanism of lateral collision and the kinematic model of vehicles. Owing to their simplicity, precision, and ease of comprehension, these parameter-based methodologies serve as competent risk assessment methods for single-lane scenarios. Nevertheless, their adaptation to multilane traffic environments is restricted because they typically only consider the one-dimensional longitudinal motion of vehicles.

The artificial potential field (APF) theory was initially implemented for collision-free trajectory planning in robotics by Khatib [16], evidencing its robust path-planning capabilities. To resolve the issue of the applicability of the TTC model in a multidimensional traffic environment, the APF theory was extended to the realm of driving risk assessment. Huang et al. employed a long short-term memory (LSTM) network to devise an intention identification model and risk assessment model, both anchored in the driving safety field [17]. These two models were subsequently integrated to facilitate a comprehensive risk assessment. In a recent study, Wang et al. [18] merged APF with vehicular kinematics and dynamics and introduced a unified quantitative method based on the equivalent force model. A comprehensive driving risk model was constructed considering the trifecta of driver-vehicle-road (DVR) factors. Additional studies involving risk assessment were performed by the team. Huang et al. [19] developed a DVR model. This model encapsulates the implications of DVR interactions on traffic safety by integrating potential accident outcomes, unpredictable vehicle behavior, and risk sensitivity. These findings corroborate the conventional risk indicators. Furthermore, valuable data leading to improved risk assessment efficiency can be obtained from driver-attitude surveys [2].

The findings from risk assessments can facilitate delineating low-risk driving zones or supply essential data for plotting safe driving trajectories for autonomous vehicles [20, 21]. For driver-assisted vehicles, the imminent risks identified by the assessment model are typically communicated to drivers via alert mechanisms, such as audio-visual warnings, or solicited by active interventions enabled by AEB systems [22, 23] or automatic emergency steering [24]. Simultaneously, the results of scientific risk assessment provide the necessary basis for formulating relevant policies [25].

Based on the analysis of existing research, the following issues exist in the study of collision risk assessment: (1) some models are applicable only to the longitudinal motion of vehicles and ignore the influence of adjacent-lane vehicles. (2) Some studies overlooked the impact of future changes in vehicle motion trends on driving risks. (3) Almost all studies focused on the likelihood of a crash and ignored the impact of crash severity on risk.

This study emphasizes the crucial role of risk assessment in traffic safety and autonomous driving planning, highlighting the current scarcity of related research. It focuses on developing a more rational and scientific approach to assessing vehicle crash risks. A methodology that uses collision probability and intensity to characterize the pre- and post-crash risk indicators, respectively, is proposed. A drone-based vehicle trajectory dataset, CitySim [26], is used to simulate vehicle perception of the surrounding driving environment in the vehicle-to-vehicle (V2V) context. This simulation includes attributes of surrounding vehicles such as speed, acceleration, and location. The vehicles in the scene are categorized into one target vehicle and multiple background vehicles according to the different objects of the study. The traveling trajectories of the two vehicles are predicted and generated as the basis for the collision risk assessment model.

The remainder of this paper is organized as follows. The Methodology section presents the proposed trajectory and collision risk assessment models. The Experiment, Results and Discussion section offers the validation of the proposed model using publicly available data. The Conclusions section presents the conclusions, limitations, and directions for future work.

## Methodology

### Overall framework

**Fig 1:**
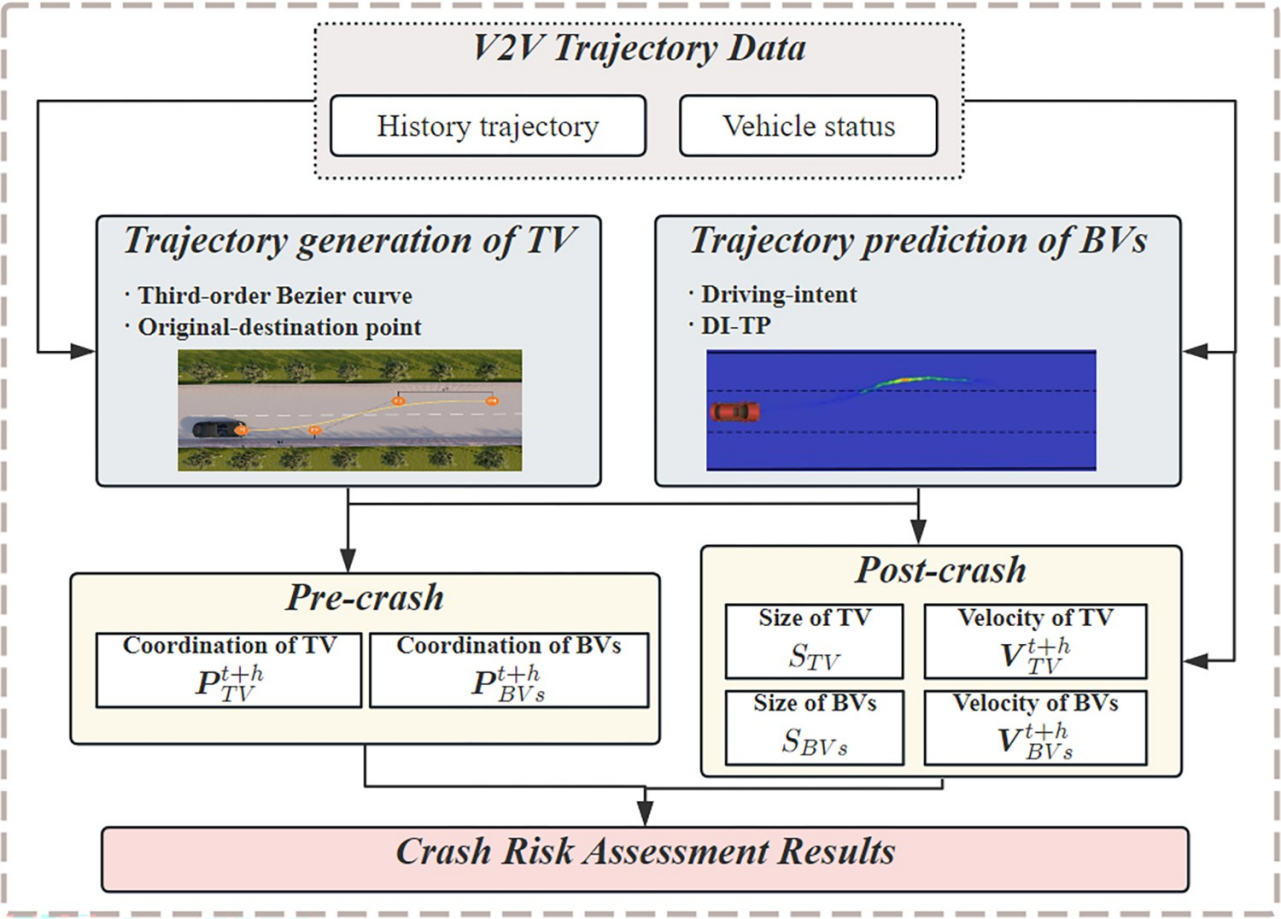
Overall framework.

In this study, vehicles within the scenario were classified into two groups: a randomly chosen target vehicle (TV) and background vehicles (BVs). The scenario includes only one TV, which can be any vehicle, while the BVs encompass all vehicles apart from the TV. This scenario closely resembles heterogeneous mixed traffic comprising autonomous vehicles and human-operated vehicles. The comprehensive framework for crash risk assessment is illustrated in Fig 1. The attributes and trajectory data of vehicles were obtained by simulating a V2V environment. Then, the trajectories of the TVs and BVs were analyzed separately; a trajectory prediction model was proposed to predict the trajectories of the BVs, and third-order Bezier curves were used to generate the candidate TV trajectories.

### V2V Trajectory data

The ICV can perceive the driving environment and obtain the driving status of surrounding vehicles through onboard sensors such as cameras, radars, and LiDARs. It can transmit critical information to other vehicles via V2V communication. Thus, for a given scenario, the following information is available:

$$\left\{ \begin{array}{c} S(t,r)=[V_{1},V_{2},\cdots ,V_{n}]\\ V_{i}=[z_{1},z_{2},\cdots {,z}_{m}],(i=1,2,\cdots ,n)\\ z_{m,n}=[z_{m,n}^{t-t_{h}},z_{m,n}^{t-t_{h}+1},\cdots ,z_{m,n}^{t},z_{m,n}^{t+1},\ldots ,z_{m,n}^{t+t_{p}-1},z_{m,n}^{t+t_{p}}] \end{array}\right.$$ (1)

**Fig 2:**
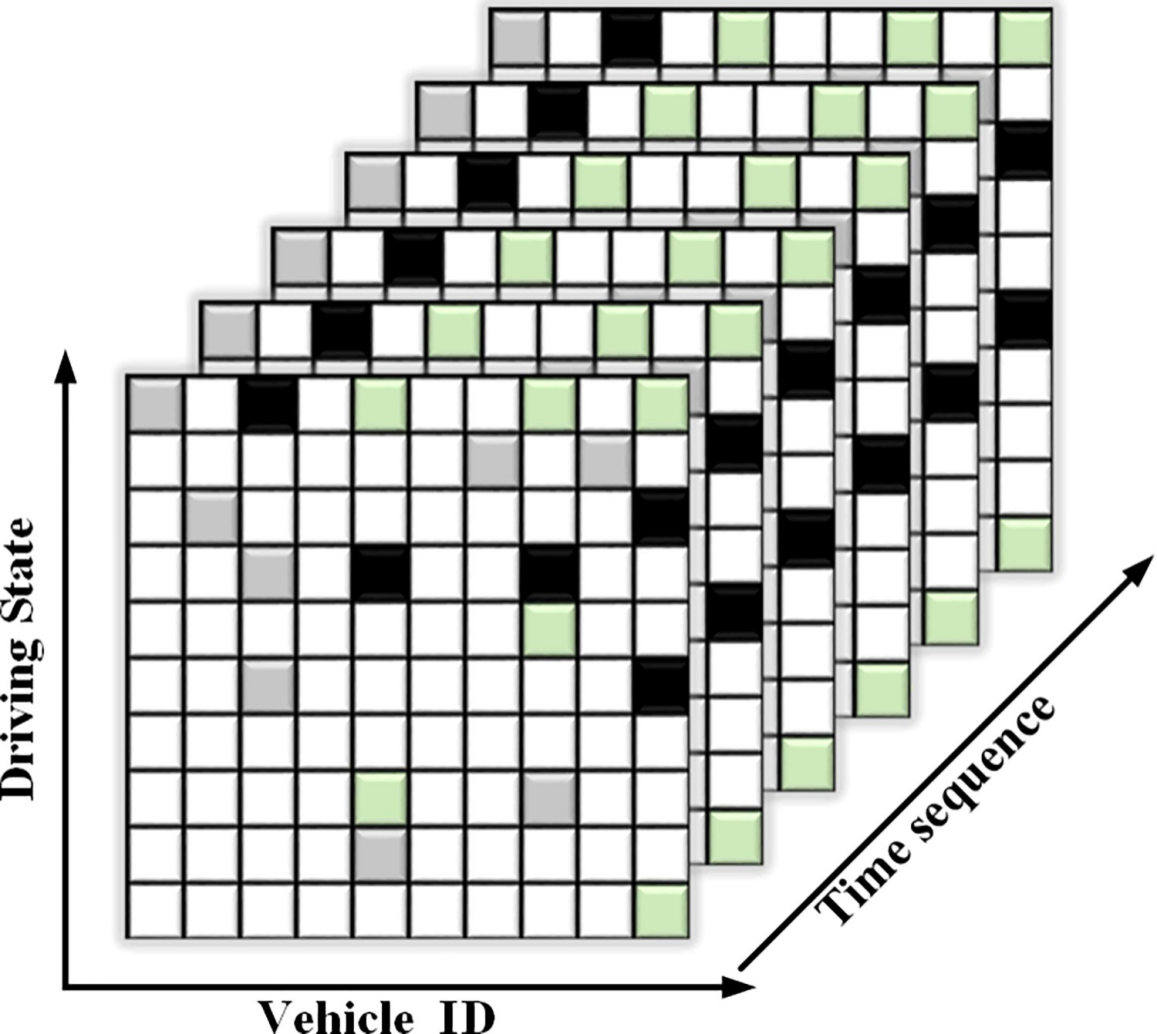
3D matrix structure of V2V trajectory data.

*S(t*, *r)* represents a scenario with time *t* and range *r*. *V*_*i*_ represents the various vehicles in the scenario ***z***_*m*_ is the vehicle data pertinent to evaluating the driving risk, including the trajectory and vehicle driving status. Because vehicles moved over time, the *m*-dimensional parameter of the vehicle *n* could be described as ***z***_*m*,*n*_, and [*t*-*t*_*h*_, *t*] and [*t*, *t*+*t*_*p*_]are past or future moments. The V2V trajectory data can also be represented using a 3D matrix, as shown in Fig 2, where the three axes represent different vehicle IDs, time nodes, and driving states.

Jia et al. [27] constructed a model that amalgamated game theory with deep learning, relying on the historical driving states of vehicles in a given scenario. The hybrid model achieved a prediction accuracy of 94.56% for standard driving behaviors (left lane-changing, right lane-changing, lane-keeping, acceleration, and deceleration) in urban expressway scenarios within a time span of 0.2–0.4 s. In a defined scenario, perceiving the future driving intentions of all vehicles is feasible. The perception outcome for vehicle *n* at time *t* can be expressed as:

$$z_{n,intent}^{t}=[\lambda_{LC},\lambda_{RC},\lambda_{LK},\lambda_{A},\lambda_{D},\lambda_{S},]$$ (2)

where $z_{n,intent}^{t}$ is the driving intent of vehicle *n* at time *t*, *λ*∈[0,1] represents the distribution probability of different driving intents, with *λ*_*LC*_, *λ*_*RC*_, *λ*_*LK*_, *λ*_*RA*_, *λ*_*RD*_, and *λ*_*S*_ representing the six typical driving intents: left lane-changing, right lane-changing, lane keeping, acceleration, deceleration, and stopping, respectively.

### Trajectory prediction for BVs

Traditional trajectory prediction focuses on extracting the distribution of historical trajectories and using a model that infers the distribution of future trajectories of a vehicle in the subsequent seconds. The trajectory of a vehicle is closely related to its driving intention. For example, if a vehicle has more than 90% intention to change lanes to the right lane, the future trajectory should be concentrated in the right lane, and its probability of being in the other lane should be much lower. Therefore, in predicting BV trajectories, combining the information on driving intention and the current driving environment can help reduce the influence of low-probability trajectories on the overall prediction accuracy and narrow the output trajectory space, which is vital in improving the trajectory prediction accuracy.

**Fig 3:**
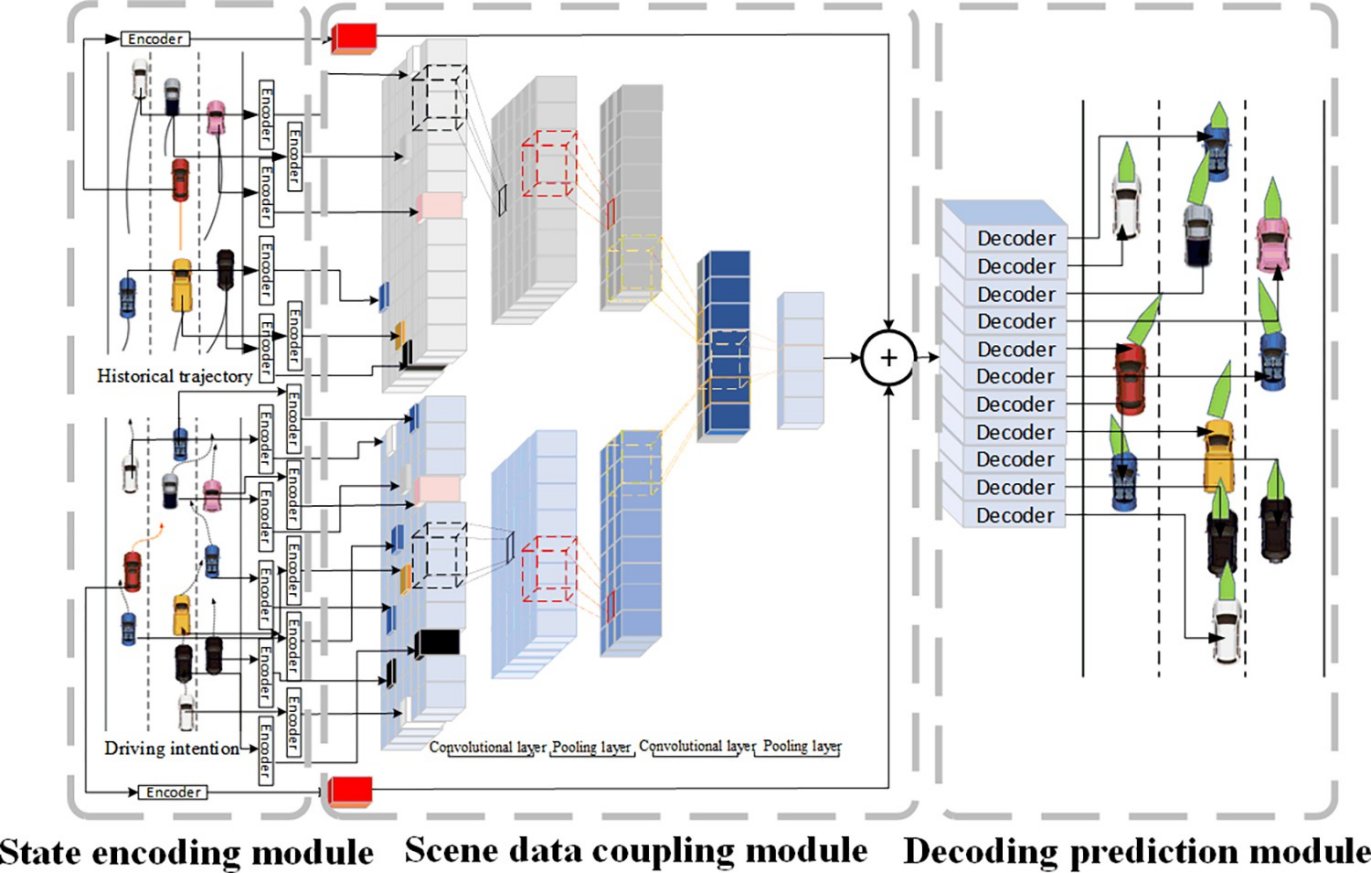
Structure of DI-TP model.

Following this principle, a driving-intention-based trajectory prediction model (DI-TP) was proposed to accurately predict the trajectories of BVs. The structure of the model is illustrated in Fig 3. The DI-TP model structure includes three main components: the state-encoding, scene-data coupling, and decoding-prediction modules. The input data to the model are historical trajectories (mainly containing coordinates, velocity, and acceleration) and the driving intentions of vehicles within a specific range. The output is the probability distribution of the trajectories of future moments.

### State-encoding module

The primary role of the state-encoding module is to remap the input sequence into a fixed-length vector following a predetermined schema and extract the kinematic features of the vehicle and temporal attributes from the data. The input data for this module were divided into historical trajectory data and driving intention data.

$$\left\{ \begin{array}{c} I=[z_{tr,n},z_{v,n},z_{a,n},z_{DI,n}^{t}]\\ \frac{z_{tr,n}=[(x,y{)}_{n}^{t-t_{h}},(x,y{)}_{n}^{t-t_{h}+1},\cdots ,(x,y{)}_{n}^{t-1},(x,y{)}_{n}^{t}]}{z_{v,n}=[v_{n}^{t-t_{h}},v_{n}^{t-t_{h}+1},\cdots ,v_{n}^{t-1},v_{n}^{t}]}\\ \frac{z_{a,n}=[a_{n}^{t-t_{h}},a_{n}^{t-t_{h}+1},\cdots ,a_{n}^{t-1},a_{n}^{t}]}{z_{DI,n}^{t}=[\lambda_{LC},\lambda_{RC},\lambda_{LK},\lambda_{A},\lambda_{D},\lambda_{S}]} \end{array}\right.$$ (3)

where ***I*** is the input data, ***z***_*tr*,*n*_, ***z***_*v*,*n*_, ***z***_*a*,*n*,_ and *z*^*t*^_*DI*,*n*_ are the historical trajectory, velocity, acceleration, and driving intentions, respectively, and [*t-t*_*h*_, *t*] represents the past period.

The state-encoding module was implemented using an LSTM encoder, which augments a conventional recurrent neural network. At each time *t*, the most recent historical state and driving intention data segment were extracted as features and then outputted as a fixed vector via the LSTM encoder. The vectors corresponding to each vehicle undergo iterative updates frame-by-frame in the past frame data, wherein all LSTM encoders share weights.


$$h_{n}^{e}(t),o_{n}^{e}(t)=LSTM(I(t),h_{n}^{e}(t-1),o_{n}^{e}(t-1);W^{e})$$ (4)

The above equation is a simple representation of the coding process, where $h_{n}^{e}(t)$ and $o_{n}^{e}(t)$ are the outputs of the LSTM representing the hidden state and output vector, respectively. ***W***^*e*^ is the weight matrix of the network.

### Scene-data coupling module

Using the LSTM as an encoder enables extracting dynamic motion from the motion state information of a vehicle. Nevertheless, this dynamic information only incorporates the data tensors of an individual vehicle; in other words, every vehicle is perceived as an isolated entity, and their mutual interactions and dependencies remain unextracted. Alahi et al. [28] introduced the concept of a social tensor to express the interaction between pedestrians and their surrounding environment during movement. Building on this concept, the interactions between vehicles were mined.

Data tensors from all vehicles in the scene (excluding the vehicle under prediction) were input into the social tensor to compile a social tensor pool. At this time, all information that potentially impacts the operating state of the vehicle under prediction was housed within the social tensor pool.

The social tensor pool can be simplified as:

$$H_{n}^{t}=\sum_{i\in N}f_{l}(z_{m,n}^{t})h_{j}^{t-1}$$ (5)

where $H_{n}^{t}$ represents the social tensor pool at time *t*. *N* is the set of vehicles, $f_{l}(z_{m,n}^{t})$ is to determine the range of vehicles that may be interacting, and $h_{j}^{t-1}$ represents the tensor data of vehicle *j* encoded by LSTM at time *t*-1. Subsequently, two successive convolution and pooling operations are performed on $H_{n}^{t}$ to obtain output vector $O_{n}^{t}$.

$$\left\{ \begin{array}{c} H_{\left(1\right)}=ReLU({Conv2D}_{\left(1\right)}(H_{n}^{t};W_{\left(1\right)}))\\ \frac{M_{\left(1\right)}=MaxPooling2D(H_{\left(1\right)})}{H_{\left(2\right)}=ReLU({Conv2D}_{\left(2\right)}(M_{\left(1\right)};W_{\left(2\right)}))}\\ M_{\left(2\right)}=MaxPooling2D(H_{\left(2\right)}) \end{array}\right.$$ (6)

where ***H***_(1)_, ***M***_(1)_, ***H***_(2)_, ***M***_(2)_ are the vectors after one and two convolutions and pooling, respectively, *ReLU*(∙) is activation function, *Conv*2*D*_(1)_(∙), *Conv*2*D*_(2)_(∙), and *MaxPooling*(∙) represent the convolution and pooling operations, respectively. ***W***_(1)_ and ***W***_(2)_ are weight matrices.

### Decoding-prediction module

The decoding-prediction module also uses the LSTM algorithm model.


$$\left\{ \begin{array}{c} O=M_{\left(2\right)}⊕FC(h_{n}^{e}(t),o_{n}^{e}(t))\\ h_{n}^{d}(t),o_{n}^{d}(t)=LSTM(O{,h}_{n}^{d}(t-1),o_{n}^{d}(t-1);W^{d}) \end{array}\right.$$ (7)

Here, ***O*** is the integrated output vector, $h_{n}^{d}(t),o_{n}^{d}(t)$ are the hidden state vector and output vector of the decoder, respectively, and ***W***^*d*^ is the weight matrix.

To describe the predicted vehicle state at a future time $z_{tr,n}=[(x,y{)}_{n}^{t+1},\cdots ,(x,y{)}_{n}^{t+t_{p}-1},(x,y{)}_{n}^{t+t_{p}}]$, this study deploys a probability distribution methodology symbolized by a Gaussian distribution with a mean value *μ*_*i*_, standard deviation of *σ*_*i*_, and correlation coefficient of *ρ*_*i*_. It is expressed as:

$$\left(x,y{)}_{n}^{t+T}∼Ν(\mu_{i}^{t+T},\sigma_{i}^{t+T},\rho_{i}^{t+T}\right)$$ (8)

The use of Gaussian distribution here helps to provide uncertainty estimates about the predicted location. This means that in addition to providing the most likely location, a probability range can also be provided to indicate the likelihood of the vehicle appearing within this range.

### Local trajectory planning for TV

The objective of TV trajectory planning is to ascertain the future driving risks of vehicles. To ensure rationality and scientific validity in risk assessment, emphasis was placed on short-term or local driving trajectories during planning. This study uses a third-order Bezier curve to simulate the shape of vehicle driving trajectories, offering superior smoothness compared to lower-order curves. In addition to the start and end points, the shape of the generated vehicle trajectory was dictated by two control points, rendering it more consistent with the actual driving trajectory of the vehicle. A third-order Bezier curve is characterized by four control points: (*X*_0_, *Y*_0_), (*X*_1_, *Y*_1_), (*X*_2_, *Y*_2_), and (*X*_3_, *Y*_3_), defined by the following equations:

$$X(t)=\sum_{i=0}^{3}X_{i}B_{i,n}(t),t\in [0,1]$$ (9)

$$X(t)=X_{0}(1-t{)}^{3}+3X_{1}t(1-t{)}^{2}+3X_{2}t^{2}(1-t)+X_{3}t^{3}$$ (10)

$$Y(t)=\sum_{i=0}^{3}Y_{i}B_{i,n}(t)$$ (11)

$$Y(t)=Y_{0}(1-t{)}^{3}+3Y_{1}t(1-t{)}^{2}+3Y_{2}t^{2}(1-t)+Y_{3}t^{3}$$ (12)

The control points of the Bezier curve are determined by the starting and ending points of the candidate trajectories and driving intentions. The curvature of any point on the curve can be calculated using Eq (13):

$$K(t)=\frac{1}{\rho (t)}=\frac{X^{\prime }(t)Y^{\prime \prime }(t)-X^{\prime \prime }(t)Y^{\prime }(t)}{(X^{\prime 2}(t)Y^{\prime 2}(t){)}^{3/2}}$$ (13)

In order to ensure the effectiveness and accuracy of the planned trajectory. The trajectory planning of the target vehicle is carried out on the basis of driving intention perception, which on one hand reduces the computational amount of trajectory generation, and on the other hand ensures that the accuracy will not be affected by other non-existent intentions. In addition, 50 potential trajectories are generated for the target vehicle to achieve a refined coverage of the whole lane.

### Risk assessment

This section introduces a method for building a driving risk field that enables connected vehicles to evaluate collision risks with TVs. In this field, each vehicle is perceived as a potential obstacle. Risk is the combination of the probability of collision in the pre-crash period and the intensity of collision in the post-crash period. There is a risk relationship between each vehicle within a specific range, with the magnitude of risk determined by future trajectories and vehicle attributes.

$$F_{i,j}^{t}=E_{i,j}^{t}P_{t}(i,j)$$ (14)

where $F_{i,j}^{t}$ represents the risk field of vehicle *j* for vehicle *i* at time *t*; $E_{i,j}^{t}$ is the collision intensity when vehicle *i* collides with vehicle *j*; and *P*(*i*, *j*) is the collision probability between the two vehicles. There are often multiple related vehicles for a specific vehicle *i* in a scenario. Therefore, for vehicle *i*, the risk is the superposition of the risks caused by all the surrounding vehicles (represented as set *N*), which can be expressed as

$$F_{i}^{t}=\sum_{j\in Ν}E_{i,j}^{t}P_{t}(i,j)$$ (15)

The collision intensity between two vehicles stems from their characteristics as non-entirely elastic collisions, implying an energy loss that dissipates as heat and material deformation, among others. According to the physical laws, the collision intensity of two objects is dictated by their masses and speeds. While determining the mass of a vehicle remains challenging, data such as model, length, and width are more readily available, allowing for the substitution of vehicle size for weight in kinetic energy calculations. The expression is as follows:

$$E_{i,j}^{t}=k(L_{i}W_{i}{)}^{2/3}[\frac{(L_{j}W_{j}{)}^{2/3}}{(L_{i}W_{i}{)}^{2/3}+(L_{j}W_{j}{)}^{2/3}}{]}^{3}{\left|v_{i}-v_{j}\right]}^{2}$$ (16)

where *k* is the set coefficient; *L* and *W* represent the length and width of the vehicle, respectively; and *v* represents the driving speed of each vehicle. The following equation can express the running state of TV and BVs at a future moment:

$${\hat{V}}_{j}^{t+1}(z_{n}^{t+1})∼Ν(\mu_{j}^{t+1},\sigma_{j}^{t+1},\rho_{j}^{t+1})$$ (17)

The position $\left({\hat{x}}_{i}^{t+1},{\hat{y}}_{j}^{t+1}\right)$ and speed ${\hat{v}}_{i}^{t+1}$ of the target vehicle at a future moment are determined by trajectory planning. Therefore, the collision probability between two vehicles at time *t*+1 can be expressed as follows:

$$P_{t+1}(i,j)=N(a(\sqrt{({\hat{x}}_{i}^{t+1}-{\hat{x}}_{j}^{t+1}{)}^{2}({\hat{y}}_{i}^{t+1}-{\hat{y}}_{j}^{t+1}{)}^{2}}{)}^{b}|\mu_{j}^{t+1},\sigma_{j}^{t+1},\rho_{j}^{t+1})$$ (18)

where *a* and *b* are the preset parameters used to represent the relationship between the relative distance and collision probability between vehicles. By substituting Eqs (16) and (18) into Eq (14), the risk field strength of vehicle *j* for vehicle *i* can be obtained. The magnitude of the field strength reflects a dangerous situation in the environment in which the connected vehicle is located.

## Experiment, results and discussion

### Experimental data

In the risk assessment verification stage, the research was based on the perception of the driving state of the connected vehicles in the scenario. Consequently, experimental setups involving fewer than five vehicles are insufficient for effectively simulating interactions and influences between the TV and BVs. To address this, the study utilized CitySim, an open-source vehicle trajectory dataset derived from drone recordings, for validation. In addition to the increased volume of vehicles involved in the experiment, the vehicle driving status data gathered through drones efficiently resolves issues related to obstacle occlusion and communication that are typically encountered with roadside or onboard data collection equipment. CitySim includes vehicle trajectories derived from drone footage, which spans 1140 min, captured from 12 distinct locations [26]. Utilizing this dataset provides several advantages for model validation: 1) the perception of the driving state of all vehicles in the scenario is achieved, including both vehicle position and kinematic information; 2) the scope to expand driving intention data through analysis of individual vehicle driving data is offered; 3) the perception of vehicle size and type is achieved, supporting the analysis of driving risks and calculation of collision intensities; and 4) more aggressive vehicle interaction scenarios, such as weaving sections, is included, which lends more credibility and importance to risk assessment.

### BV trajectory prediction results

The actual value of the trajectory to be predicted was recorded as *y* = {*y*_1_, *y*_2_,⋯*y*_*n*_} and the predicted value of the model as $\hat{y}=\{{\hat{y}}_{1},{\hat{y}}_{2},\cdots {\hat{y}}_{n}\}$. To assess the prediction accuracy of the model, the discrepancy between predicted and actual values was computed. Because the output from the model predicting background vehicle trajectories appears as a Gaussian probability distribution of future trajectory points, the root mean square error (RMSE) and negative log likelihood (NLL) were employed as evaluation metrics. The calculations for these metrics are as follows:

$$RMSE=\sqrt{\frac{1}{n}\sum_{i=1}^{n}({\hat{y}}_{i}-y_{i}{)}^{2}}$$ (19)

$$NLL=-\sum_{i=1}^{n}\log(P(x^{i};\theta ))$$ (20)

The CitySim dataset provides the XY coordinates of the center of the vehicle, which were used as the positioning and trajectory coordinates of the system. The journey of each vehicle was segmented into fragments, each 6 s long. In these time slices, the initial 3 s represent the historical trajectory of the vehicle—the input data for the model—while the latter half serves as the predicted trajectory. This forecast was subsequently compared with the actual trajectory from the final 3 s of the sample for verification. A total of 13,000 trajectory samples were split into datasets, including six typical intentions. Lane-keeping behavior accounted for the majority of the samples, and the other five behavioral samples were almost equal in proportion. Of the samples, 70% were randomly assigned to train the model, and 30% were used for testing and evaluation.

The algorithm model employed the Adam optimizer with an established learning rate of 0.001. The LSTM models for encoding and decoding were set to 64 and 128 hidden states, respectively. The batch size was 256. Using the PyTorch framework, the deep learning model was built on a workstation with two NVIDIA RTX3080 GPUs.

Models with good experimental results for trajectory prediction in recent years were selected for comparative experiments:

*STAM-LSTM* [29]: Spatio-temporal attention LSTM model for spatio-temporal series problems.

*PiP* [30]: Planning-informed trajectory prediction for autonomous driving.

*CS-LSMT* [31]: Convolutional social pooling for vehicle trajectory prediction.

Table 1 presents the quantitative comparison results between the DI-TP model proposed in this study and other models. The DI-TP model outperformed the other methods regarding the RMSE and NLL metrics in the 1–3 s prediction range. The level of inaccuracy escalates consistently as the scope of foresight broadens.

**Table 1:** Prediction results of different models.

Indicators	Prediction range	STA-LSTM	PiP	CS-LSTM	DI-TP (this study)
**RMSE (m)**	1	0.22	0.31	0.25	0.12
	2	0.51	0.72	0.66	0.32
	3	0.82	1.33	1.19	0.68
**NLL**	1	0.34	0.53	0.62	0.28
	2	0.89	1.84	0.92	0.64
	3	1.491	2.26	1.77	1.34

In the candidate trajectory generation experiments for TVs, differences in driver habits based on various lane positions were considered. For each TV with differing driving intentions, 50 candidate trajectories in the same lane were generated.

Real vehicular scenarios from the CitySim dataset were used to validate the risk assessment effect. Several characteristic scenarios, including weaving, merging, and freeway segments, were carefully selected. These scenes were recreated based on the driving-state data for each vehicle in the dataset. Subsequently, potential risk situations for the target vehicle in these scenarios were computed and predicted.

### Risk assessment based on scenes

#### Scene 1: Weaving segment

**Fig 4:**
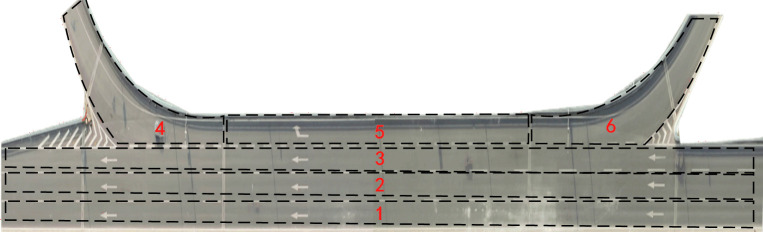
Road configurations of weaving segment.

Weaving segments, marked by frequent lane changes and merges and accompanied by high traffic volumes and close following distances, lead to extensive interactions and conflicts among vehicles. Such conditions elevate the risk of accidents, classifying these as typical high-risk scenarios on urban expressways. The data for this scenario were derived from a fragment of the Expressway A section within the CitySim dataset, with road configurations as shown in Fig 4. It includes four lanes: a merging region and a diverging region.

**Fig 5:**
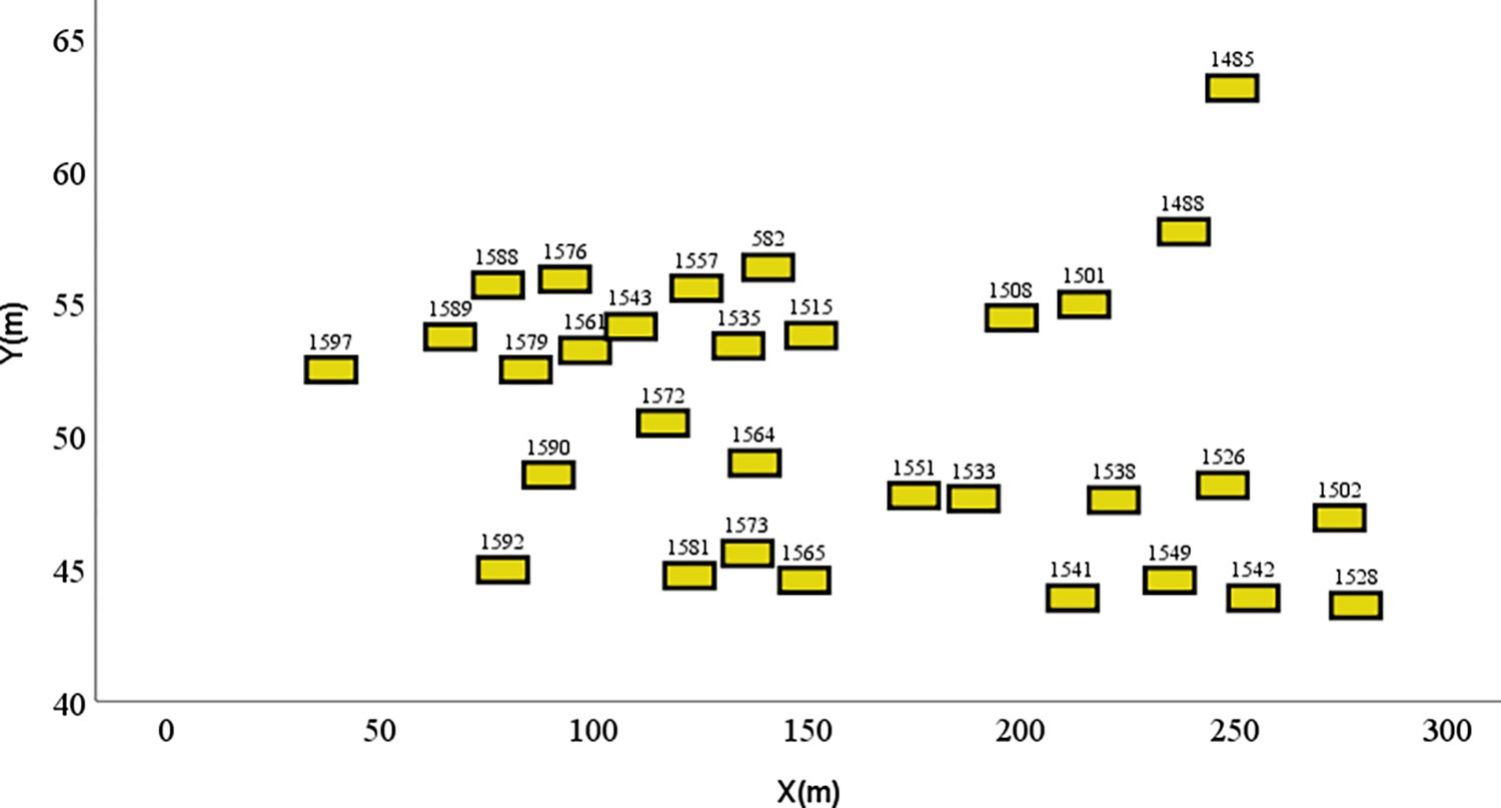
Spatial distribution of vehicles in weaving segment.

In this scenario, a random point in time was selected, with vehicle ID 1572 as the target. Fifteen other vehicles within a 50 m radius could potentially influence driving. The spatial distribution of these vehicles can be viewed in Fig 5, and the driving statuses of some vehicles in this scenario are listed in Table 2. After analyzing and calculating the intention of the vehicle, the following predictions were made: vehicle 582 is likely to remain stationary, vehicle 1515 may change lanes to the left, and the others are anticipated to maintain a steady state of motion.

**Table 2:** Vehicular statuses of the weaving segment.

Vehicle ID	X-coordinate (m)	Y-coordinate (m)	Speed (m/s)	Lane ID	Driving intention
**1572**	116.06	50.47	9.82	2	Lane-keeping
**1543**	108.53	54.10	6.39	3	Lane-keeping
**1581**	122.26	44.72	15.08	1	Lane-keeping
**1557**	123.88	55.55	5.30	5	Lane-keeping
**582**	140.68	56.33	0.00	5	Stop
**1515**	150.73	53.76	6.06	3	Left lane-changing
**……**	……	……	……	……	…………

**Fig 6:**
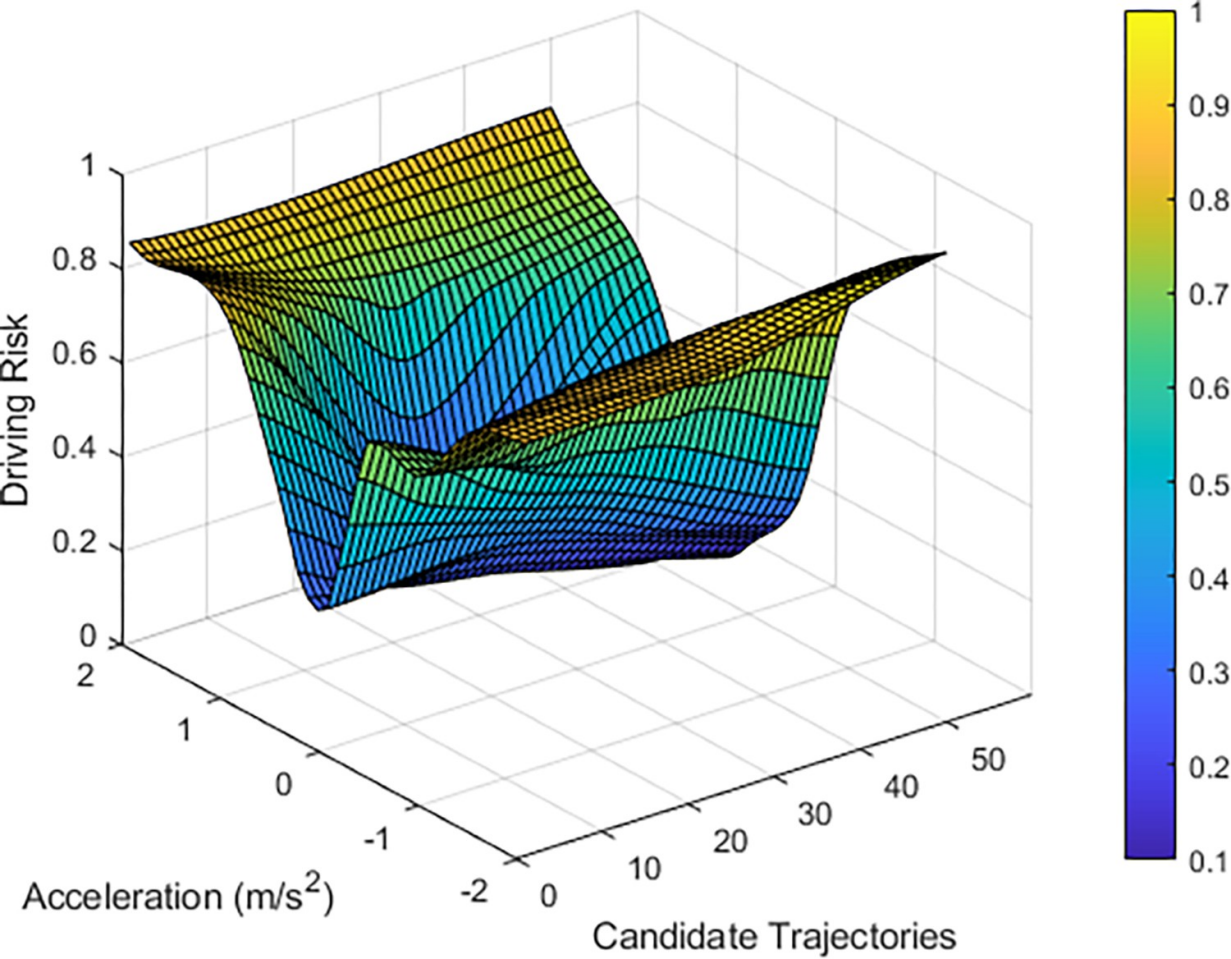
Results of the driving risk assessment for vehicle 1572.

By analyzing the trajectories of the TV and BVs, combined with the results of the driving risk assessment model, the driving risk of vehicle 1572 can be determined, as shown in Fig 6. The elevated risk status of this vehicle is primarily attributed to its dense distribution. Given the intent of vehicle 1572 to maintain its lane, reducing its speed could significantly increase its collision risk with vehicle 1590. Moreover, its trajectory, which is closely aligned with the edge of the lane, can result in collisions with other vehicles in lane 3 when considering potential trajectories.

### Scene 2: Merging segment

**Fig 7:**
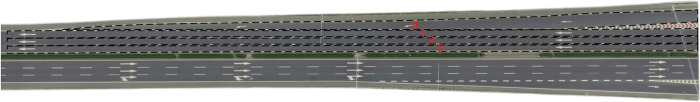
Road configurations of the merging segment.

The merging segment, which involves the convergence of multiple-lane traffic flows, is a section where lane-changing behavior is expected and can be used to study the impact of lane-changing behavior on traffic safety. The data for the merging segment in this section are sourced from the Freeway C dataset of CitySim, which comprises four one-way lanes labeled as lane 0–3. Lane 0 functions as the ramp, as depicted in Fig 7.

**Fig 8:**
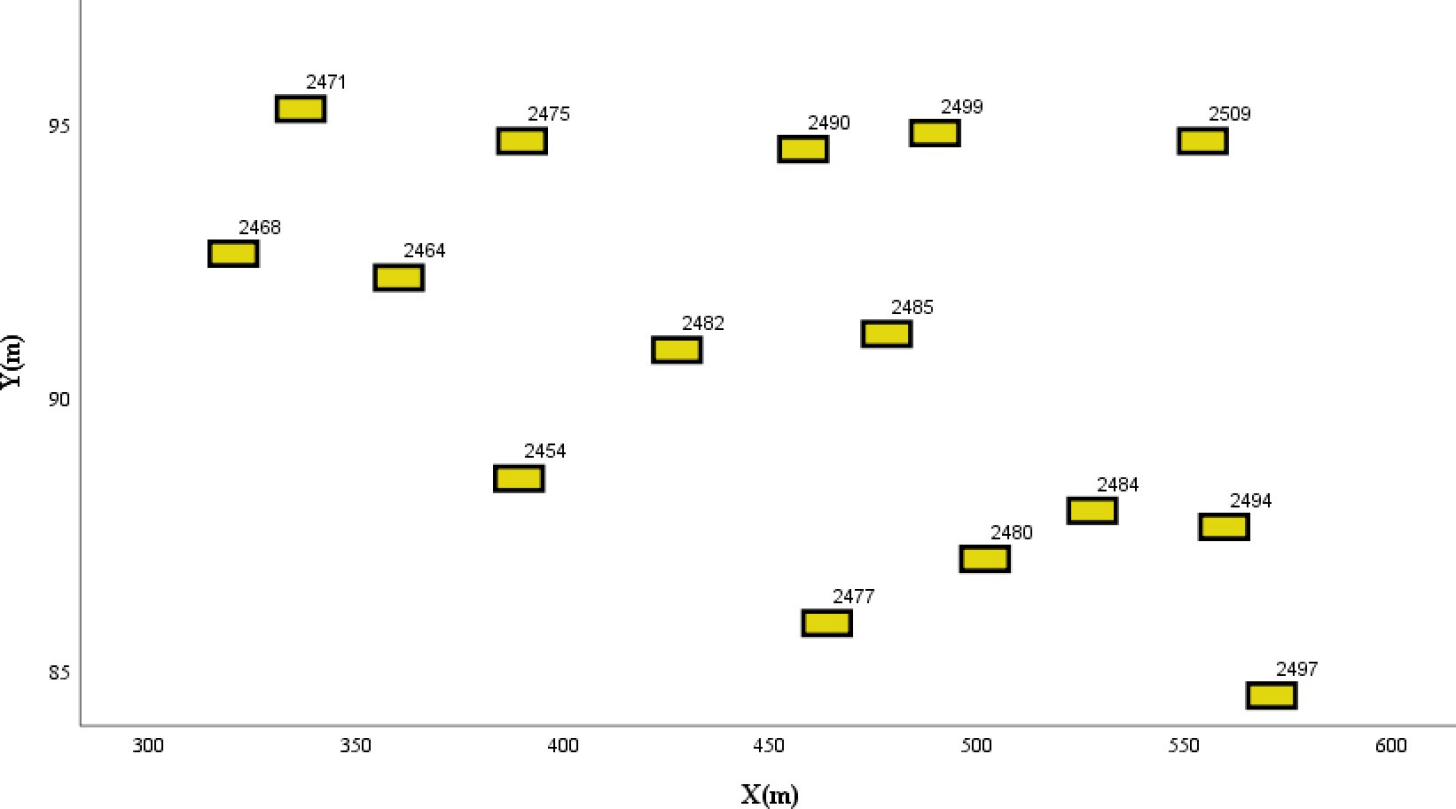
Spatial distribution of vehicles in the merging segment.

In this scenario, vehicle 2484 is designated as the TV. It enters from the ramp and maintains its trajectory in lane 1. Seven BVs are within the affected range. Fig 8 shows the distribution of each vehicle in the context of the lane-changing scenario. By analyzing the driving states of the TV and BVs, the driving intentions of the vehicles can be predicted, as shown in Table 3. Because of their current position on the ramp, vehicles 2477 and 2497 are anticipated to change lanes from lane 0 to 1 shortly. The presence of these ramp vehicles affects the driving space and safety of vehicle 2484, thus predicting its inclination to change lanes to lane 2.

**Table 3:** Vehicular statuses of the merging segment.

Vehicle ID	X-coordinate (m)	Y-coordinate (m)	Speed (m/s)	Lane ID	Driving intention
**2484**	527.80	87.94	21.73	1	Right lane-changing
**2480**	501.87	87.06	19.45	1	Lane-keeping
**2477**	463.72	85.88	22.73	0	Right lane-changing
**2494**	559.62	87.65	17.17	1	Lane-keeping
**2497**	571.11	84.55	17.56	0	Right lane-changing
**……**	……	……	……	……	…………

**Fig 9:**
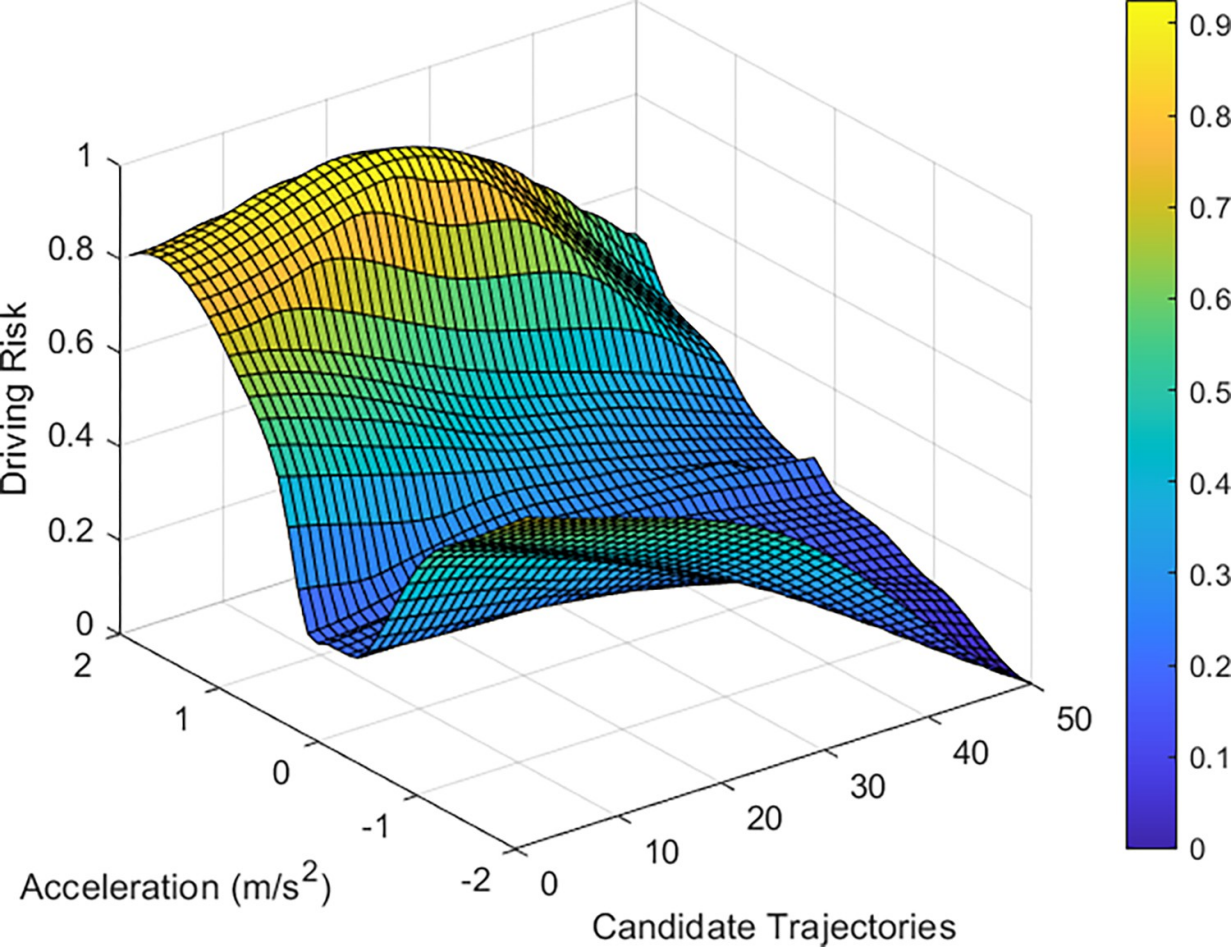
Results of the driving risk assessment for vehicle 2484.

By analyzing the vehicle driving trajectories, intentions, and statuses in the scenario, the collision risk of vehicle 2484 was evaluated using the driving risk assessment model, as shown in Fig 9. The risk distribution graph shows that lane-changing scenarios carry heightened risks, which are generally caused by increased vehicle speeds, reduced vehicle spacing, and frequent lane changes. Vehicle 2484 aims to shift to lane 2 on the right; however, its trajectory is influenced by the BVs. It faces a higher risk of collision if it accelerates and navigates closer to the left boundary of lane 2. Simultaneously, because of the lower driving speeds of the following vehicles, the collision probability of the TV is lower if it decelerates and drives to the left. These experimental results align with the vehicle distribution presented in Fig 8.

### Scene 3: Freeway segment

The freeway segment serves as a basis for evaluating the capability of the risk assessment model to analyze the collision risks for vehicles that intend to maintain their lanes longitudinally. The test scenario was developed using the Freeway B section from the CitySim dataset, featuring six straight lanes in both directions. For this study, three one-directional lanes, labeled sequentially from lane 0 to lane 2, were selected.

**Fig 10:**
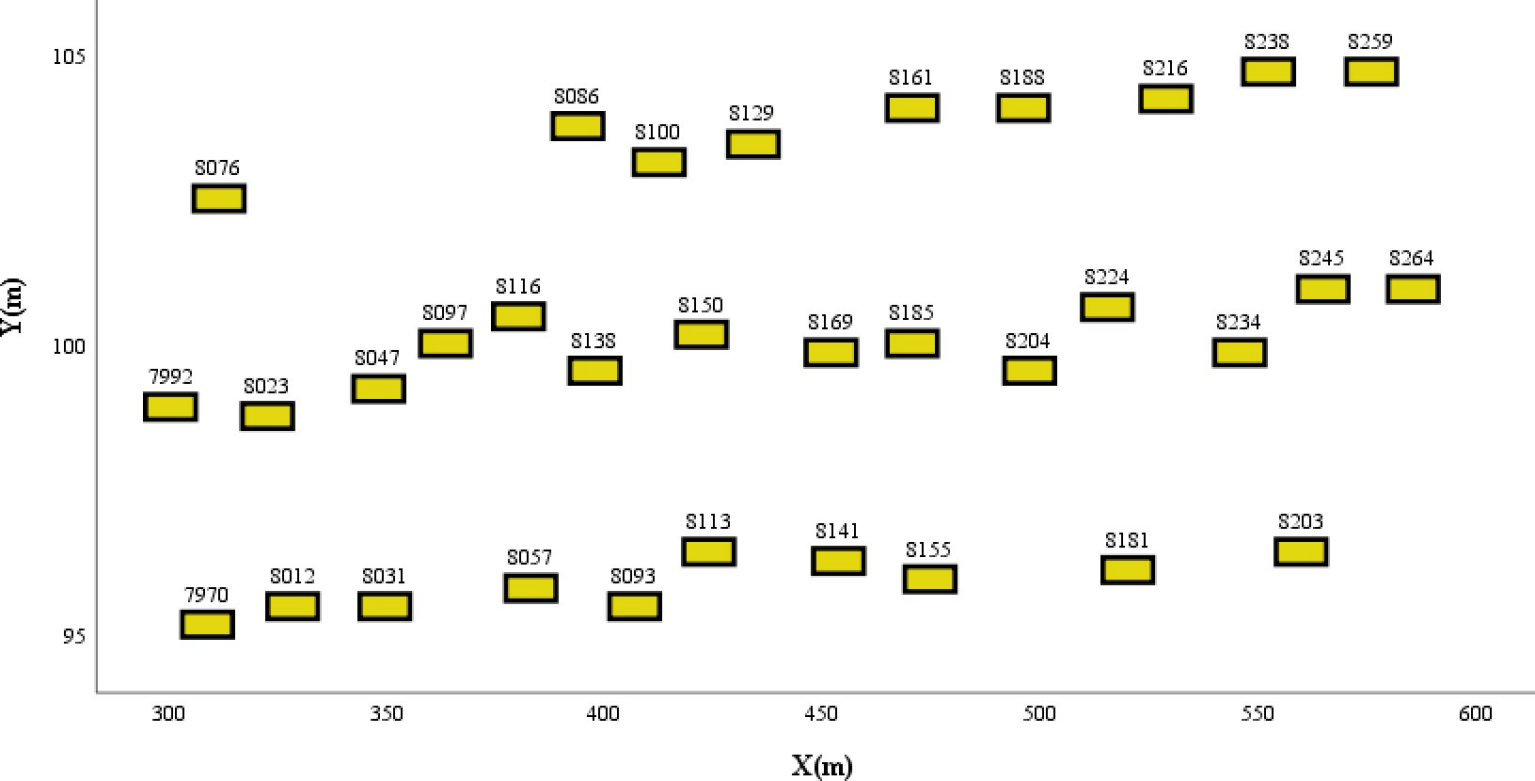
Spatial distribution of vehicles in the freeway segment.

**Fig 11:**
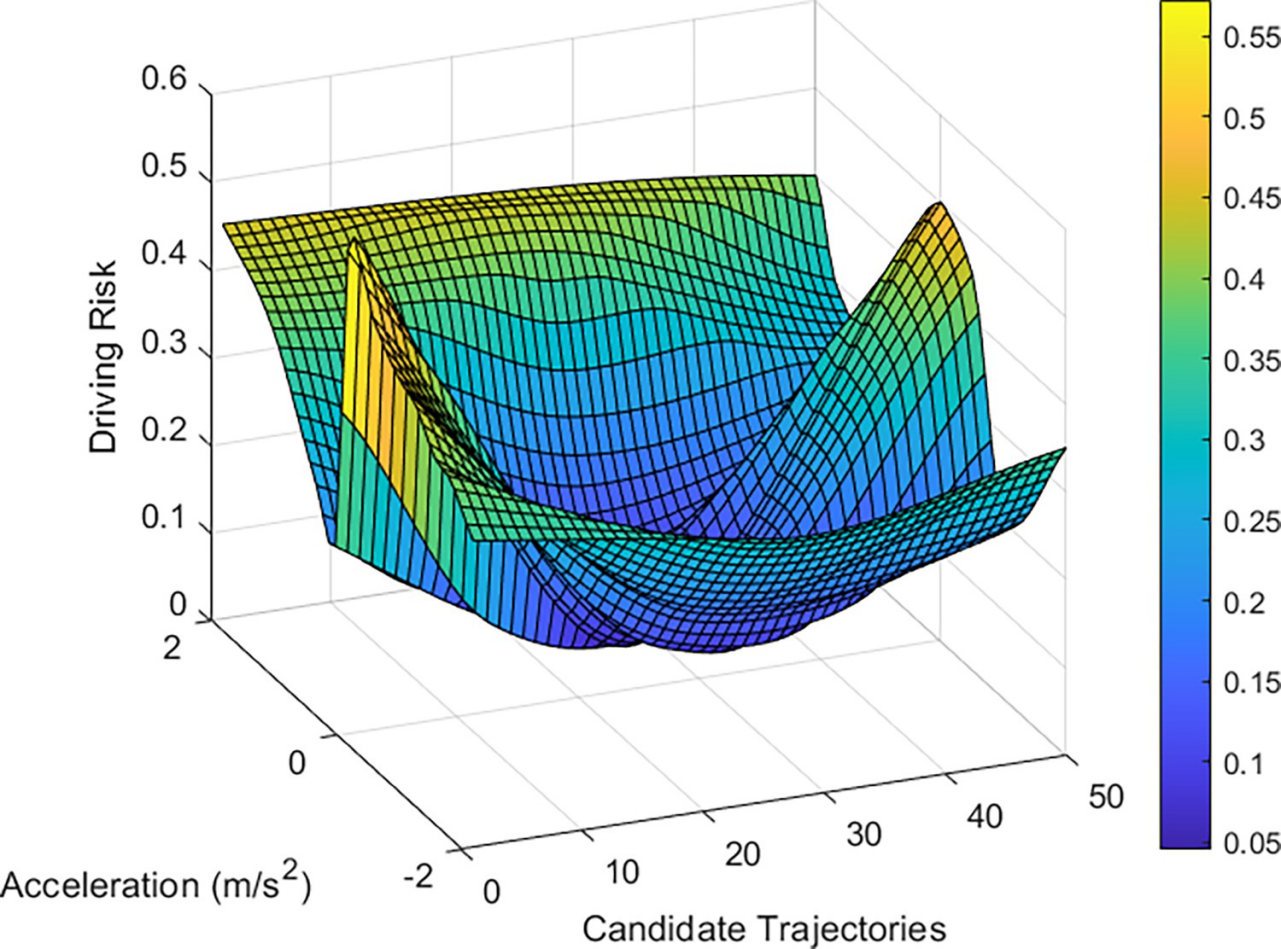
Results of the driving risk assessment for vehicle 8185.

In this scenario, most vehicles maintain their lanes while traveling at a comparably low speed, leading to a dense and steady traffic flow. Accordingly, vehicle 8185, selected randomly from the middle lane, serves to substantiate the competence of the model in determining the collision risk associated with freeway segments. The six surrounding vehicles presented potential risks to the safety of vehicle 8185. The vehicle positions in the scene are shown in Fig 10. Table 4 details the driving statuses of the selected vehicles within the scenario, with all vehicles maintaining their lanes at an average speed of approximately 15 m/s. Fig 11 shows the results of the risk assessment model. Owing to the stable traffic flow, the overall scenario was characterized by a lower likelihood of collisions. The potential risks to vehicle 8185 are mainly from abrupt acceleration or deceleration, which could lead to collisions with other vehicles. Furthermore, deviation from the center of a lane may lead to collisions with vehicles in adjacent lanes.

**Table 4:** Vehicular statuses in the freeway segment.

Vehicle ID	X-coordinate (m)	Y-coordinate (m)	Speed (m/s)	Lane ID	Driving intention
**8185**	470.57	100.03	15.72	1	Lane-keeping
**8169**	451.95	99.88	15.65	1	Lane-keeping
**8150**	422.20	100.19	16.57	1	Lane-keeping
**8129**	434.10	103.48	15.31	2	Lane-keeping
**8161**	470.57	104.10	13.15	2	Lane-keeping
**……**	……	……	……	……	…………

## Conclusions

### Summary and conclusions

Collision risk assessment of ICVs has significant implications for planning safe trajectories and ensuring driving safety. As a possible driver operation in the future, driving intention is an essential basis for risk assessment and affects the scientific accuracy of risk assessment. Based on the perception of driving intention, this study investigated vehicle driving risk assessment in an environment that simulated V2V information interaction. A driving risk assessment model based on intention perception was proposed, with the risk assessment process divided into three parts: trajectory prediction for background vehicles, candidate track planning for the target vehicle, and multi-target collision risk evaluation. The following main conclusions are based on the experimental results:

1. Incorporating the driving intention and vehicle interaction factors into the process of vehicle trajectory prediction significantly improved the accuracy of the prediction results. This finding was reflected in the significant reduction in the RMSE and NLL indicators compared with those of the other models.
2. The third-order Bezier curve employed to generate candidate trajectories ensured smoothness, continuity, and controllability. Simultaneously, considering the different driving habits of drivers is necessary to generate multiple candidate trajectories covering the entire lane, which can be used to analyze the collision risks encountered in different driving areas.
3. Assessing driving risk by combining the probability of a pre-crash event with the intensity of a post-crash event is more scientific than using TTC and APF methods.
4. This study verified the effectiveness and scientificity of the proposed risk assessment model using three traffic scenario datasets. The results of the risk assessment were generally consistent with the likelihood of a collision due to the environment of the vehicle.
5. In the weaving and merging sections, most vehicles in the entire scenario had a higher collision risk than those in the freeway section because of the frequent lane-changing behavior. This indirectly proves the importance of smooth and undisturbed traffic flow for traffic safety.

### Limitations and future work

This study assessed vehicle collision risk in a connected environment, thereby improving the scientificity and effectiveness of the assessment. However, this study had the following limitations:

1. The risk assessment process failed to consider the shape and edges of the vehicles, relying solely on the distance from the center point of the vehicle to determine the collision risk.
2. Drone-based traffic flow data differ from driving-status data gathered from actual connected vehicle interactions, overlooking potential communication delays.

In future investigations, the objective will be to establish an authentic multi-vehicle connected experimental setting with the intent of investigating collision risk assessment for intelligent networked vehicles. Simultaneously, additional factors will be taken into consideration to comprehensively assess crash risk, thereby making a substantial contribution to overall traffic safety.
